# Antihypertensive Effect in Vivo of QAGLSPVR and Its Transepithelial Transport Through the Caco-2 Cell Monolayer

**DOI:** 10.3390/md17050288

**Published:** 2019-05-13

**Authors:** Liping Sun, Beiyi Wu, Mingyan Yan, Hu Hou, Yongliang Zhuang

**Affiliations:** 1 Yunnan Institute of Food Safety, Kunming University of Science and Technology, No. 727 South Jingming Road, Kunming 650500, China; kmlpsun@163.com (L.S.); wubeiyi1994@163.com (B.W.); 2 Shandong Provincial Key Laboratory of Biochemical Engineering, College of Marine Science and Biological Engineering, Qingdao University of Science and Technology, Qingdao 266042, China; yanmingyan@qust.edu.cn; 3 Food Science and Technology, Ocean University of China, No 5, Yushan Road, Qingdao 266005, China; houhu@ouc.edu.cn

**Keywords:** QAGLSPVR, antihypertensive effect, Caco-2 cell monolayer, transport routes

## Abstract

The peptide QAGLSPVR, which features high angiotensin-I-converting enzyme (ACE) inhibitory activity, was identified in our previous study. In this study, the in vivo antihypertensive effect of QAGLSPVR was evaluated. Results showed that QAGLSPVR exerts a clear antihypertensive effect on spontaneously hypertensive rats (SHRs), and the systolic and diastolic blood pressures of the rats remarkably decreased by 41.86 and 40.40 mm Hg, respectively, 3 h after peptide administration. The serum ACE activities of SHRs were determined at different times, and QAGLSPVR was found to decrease ACE activities in serum; specifically, minimal ACE activity was found 3 h after administration. QAGLSPVR could be completely absorbed by the Caco-2 cell monolayer, and its transport percentage was 3.5% after 2 h. The transport route results of QAGLSPVR showed that Gly-Sar and wortmannin exert minimal effects on the transport percentage of the peptide (*p*> 0.05), thus indicating that QAGLSPVR transport through the Caco-2 cell monolayer is not mediated by peptide transporter 1 or transcytosis. By contrast, cytochalasin D significantly increased QAGLSPVR transport (*p*< 0.05); thus, QAGLSPVR may be transported through the Caco-2 cell monolayer via the paracellular pathway.

## 1. Introduction

Hypertension is considered a public cardiovascular disease and an important risk factor of myocardial infarction, cerebral infarction, and renal failure. Angiotensin I-converting enzyme (ACE) plays a key role in controlling hypertension. ACE inhibition is an important method often used to treat high blood pressure [1]. Previous studies show that many peptides from food materials have ACE inhibitory (ACEI) activity and decrease blood pressure [2,3]. This function of peptides has received considerable research attention.

Previous studies showed some bioactive peptides existed good antihypertensive activity in vivo [4,5]. Bioactive peptides with high ACEI activities in vitro can exert antihypertensive activity in vivo when they are absorbed intact in the target organ through the intestinal tract [6]. Although some peptides have in vitro ACEI activity, no antihypertensive activity in spontaneously hypertensive rats (SHRs) has yet been observed after oral administration in vivo. Thus, theACEI activities of peptides may be affected by the absorption and metabolismin vivo [7]. Caco-2 cells are human colon adenocarcinoma cell clones. The structures and functions of Caco-2 cells are similar to those of differentiated intestinal epithelial cells [8]. Therefore, a Caco-2 cell monolayer model is often used in simulated intestinal transport experiments in vitro. Previous studies have studied the transepithelial transports of antihypertensive peptides by Caco-2 cell monolayer model [8,9].

In our previous studies, enzymatic hydrolysates of tilapia skin gelatin were obtained using simulated gastrointestinal digestion. The hydrolysates were isolated and purified, and QAGLSPVR was obtained by successive chromatography of the gelatin hydrolysates [10]. The molecular weight of QAGLSPVR is 826.4661 Da, and its IC_50_ of ACEI activity is 68.35 μM [10]. The present study aims to confirm the in vivo antihypertensive effect of QAGLSPVR by using the SHR model. The transepithelial transport of QAGLSPVR was evaluated according to the Caco-2 cell monolayer model via an ultra-performance liquid chromatograph coupled to a Q Exactive hybrid quadrupole-orbitrap mass spectrometer (UPLC-Q-Orbitrap-MS^2^). Finally, the transport routes of QAGLSPVR in the Caco-2 cell monolayer were analyzed.

## 2. Results

### 2.1. Changes in Blood Pressure over Time

**Figure 1:**
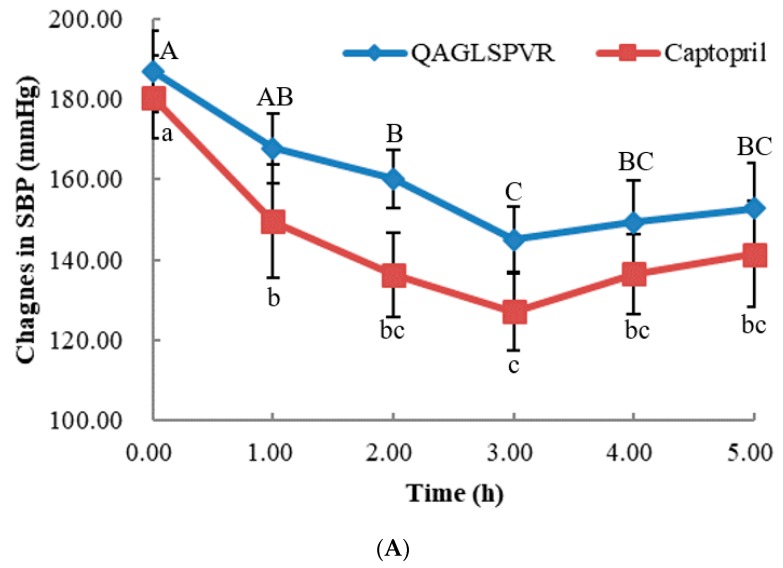

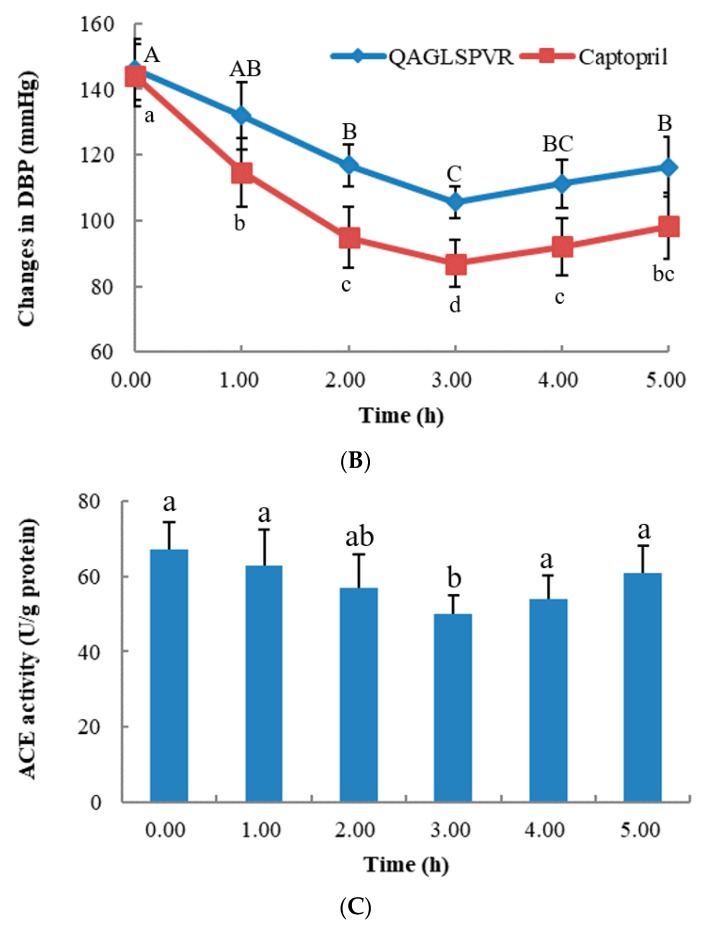
In vivo effects of 20 mg/kg BW QAGLSPVR and 10 mg/kg BW captopril on spontaneously hypertensive rats, (**A**): systolic blood pressure (SBP), different capital letters indicated significant differences for QAGLSPVR with different times and different lowercase letters indicated significant differences for captopril with different times; (**B**): diastolic blood pressure (DBP), different capital letters indicated significant differences for QAGLSPVR with different times and different lowercase letters indicated significant differences for captopril with different times; (**C**): ACE activity in serum, different letters indicated significant differences for QAGLSPVR with different times (*p* < 0.05).

As shown in Figure 1A,B, the systolic (SBP) and diastolic (DBP) blood pressure of SHRs obviously decreased after a single treatment of oral QAGLSPVR. The SBP and DBP obtained were lowest 3 h after QAGLSPVR administration. Compared with those of the control group, the SBP and DBP of the QAGLSPVR- treated group significantly decreased by 41.86 and 40.40 mm Hg (*p* <0.05), respectively, 3 h after administration. Similar results were found in the group of SHRs receiving 10 mg/kg body weight (BW) captopril serving as positive control.

ACE activities in the serum of SHRs were determined after a single oral administration of 20 mg/kg BW QAGLSPVR. As shown in Figure 1C, serum ACE activities in the QAGLSPVR group significantly decreased3 h after administration (*p* < 0.05) compared with those of the control group. Thereafter, serum ACE activities increased with time. This trend is consistent with the change in blood pressure of SHRs.

### 2.2. Transport through the Caco-2 Cell Monolayer

**Figure 2:**
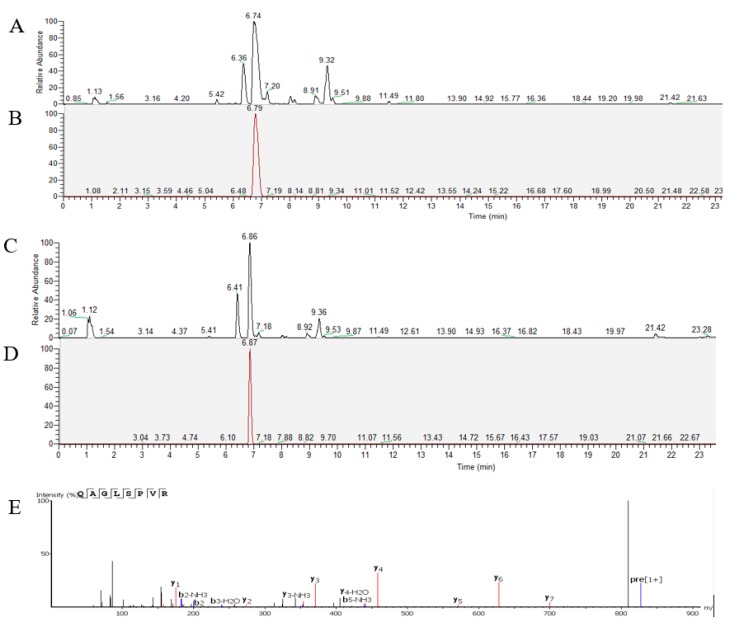
The chromatograms of QAGLSPVR as detected by UPLC-Q-Orbitrap-MS^2^, (**A**): Total ion chromatograms of apical chamber; (**B**): Extract ion chromatograms of QAGLSPVR in apical chamber; (**C**): Total ion chromatograms of basal chamber, (**D**): Extract ion chromatograms of QAGLSPVR in basal chamberand; (**E**): Identification of QAGLSPVR by *De Novo*™ software.

QAGLSPVR transport was analyzed using the Caco-2 cell monolayer model. Qualitative and quantitative analyses of QAGLSPVR were performed using UPLC-Q-Orbitrap-MS^2^. Figure 2A,B respectively show the total and extract ion chromatograms of QAGLSPVR in the apical chamber (AP) of the Caco-2 cell monolayer. Figure 2C,D respectively show the total and extract ion chromatograms of QAGLSPVR in the basal chamber (BL). QAGLSPVR identification was conducted using *De Novo*™ software (Peak Studio 7.5, Bioinformatics Solutions, Inc., Waterloo, ON. Canada) (Figure 2E).

**Figure 3:**
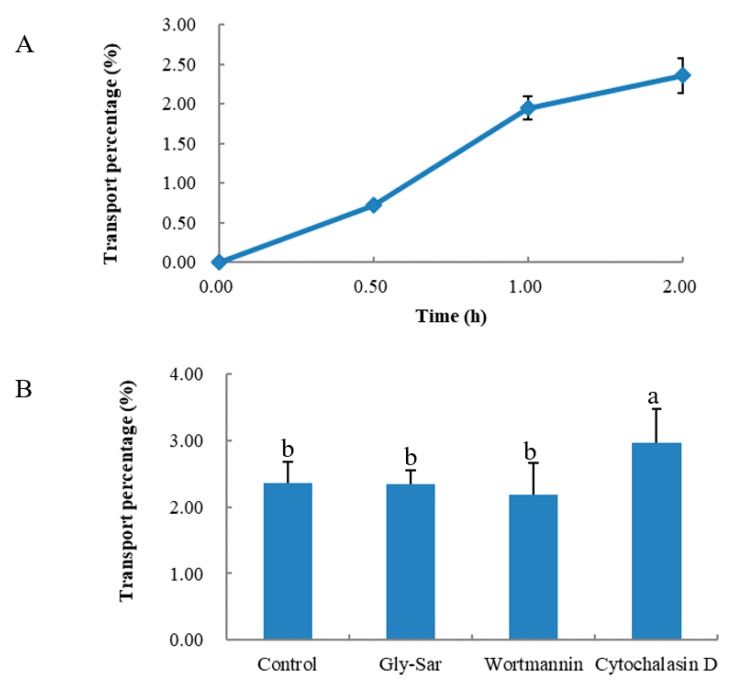
Transepithelial transport of QAGLSPVR in presence of inhibitory/disruptors for different transportation routes by Caco-2 cell monolayer, (**A**): Transport percentage of QAGLSPVR at different times, (**B**): Transport percentage of QAGLSPVR in different routes. Different letters indicated significant differences (*p* < 0.05).

As shown in Figure 3A, QAGLSPVR transport was determined at different times. QAGLSPVR could be transported intact by the Caco-2 cell monolayer, and the transport percentage increased over time. The QAGLSPVR transport percentage was 3.5% 2 h after administration.

Figure 3B shows the transport routes of QAGLSPVR in the Caco-2 cell monolayer. Gly-Sar had no significant effect on QAGLSPVR transport (*p* > 0.05), which means QAGLSPVR transport through the Caco-2 cell monolayer is not mediated by peptide transporter 1 (PepT1). Wortmannin did not significantly affect QAGLSPVR transport (*p* > 0.05), which means QAGLSPVR transport through the Caco-2 cell monolayer is not mediated by transcytosis. Finally, cytochalasin D significantly increased QAGLSPVR transport (*p* < 0.05) through the cell monolayer, thus indicating that QAGLSPVR may be transported via the paracellular pathway.

## 3. Discussion

In our previous study, QAGLSPVR was separated and identified from tilapia skin gelatin hydrolysates, and its IC_50_ for ACEI activity in vitro was found to be 68.35 μM [10]. Bioactive peptides are exposed through systemic circulation in human tissues [11]. Unfortunately, bioactive peptides may be hydrolyzed before they reach the target tissues during passage through and absorption by the small intestine. While some bioactive peptides show in vitro ACEI activity, they do not exhibit antihypertensive effects in vivo after oral administration to SHRs. For example, FKGRYYP was identified from chicken muscle hydrolysates, and its IC_50_ for ACEI activity in vitro was found to be 0.55 mM [12]; however, no antihypertensive activity of this peptide was observed after oral administration to SHRs. Therefore, bioactive peptides must resist systemic peptidase degradation prior to reaching their target sites to exert their function in vivo. The application of antihypertensive peptides is limited when they have no ACEI activity after oral administration. In this study, we confirmed the antihypertensive effect of QAGLSPVR on the SBP and DBP of SHRs after a single oral administration of the peptide. Results showed that QAGLSPVR effectively reduces the SBP and DBP of SHRs. SBP and DBP reached maximum effect 3 h after QAGLSPVR administration. This outcome is similar to the results of a number of antihypertensive peptides, such as YASGR [13] and MEGAQEAQGD [5]. The experimental results showed that the antihypertensive effect of QAGLSPVR on SHRs is consistent with its in vitro ACEI activity.

Different ACEI peptides have different metabolic pathways and tissue distributions due to their different molecular structures [14]. Serum ACEI activity plays an important role in regulating blood pressurein vivo. Therefore, ACE activities in the serum of SHRs were evaluated after QAGLSPVR administration. The results indicated that QAGLSPVR could decrease the serum ACE activities of SHRs and regulate their blood pressures. Boonla et al. reported that rice bran protein hydrolysate can regulate plasma ACE levels to decrease the blood pressures of the 2k-1c renovascular hypertensive rats [15]. The results of this previous study are similar to those of the current work.

QAGLSPVR was proven to produce a clear antihypertensive effect on SHRs in this study. To validate whether QAGLSPVR could be completely absorbed to regulate blood pressure, the transport percentage of QAGLSPVR was evaluated via the Caco-2 cell monolayer model. Caco-2 cells can be used as an in vitro model of the human intestinal epithelium due to the various brush border membrane enzymes characterizing these cells [16]. At least eight membrane enzymes are expressed by Caco-2 cells. The brush border membrane enzymes are membrane peptidases and can hydrolyze peptides into short fragments prior to absorption of peptides [9,17]. As shown in Figure 3A, QAGLSPVR could be transported intact by the Caco-2 cell monolayer, and the QAGLSPVR transport percentage was 3.5% after 2 h. Previous studies have shown that many food-derived bioactive peptides, such as QIGLF [8], TNGIIR [18], RKQLQGVN [19], and YLGYLEQ [20], could be absorbed intact through the Caco-2 cell monolayer. This finding is similar to our results.

The characteristics of peptides, including their amino acid compositions and sequences, molecular weights, hydrogen-bond capacity, charge, and hydrophobicity, play key roles in transport over the intestinal epithelium [21]. Many studies have indicated that the transport and mechanism of peptides are highly associated with their chain length [22,23]. Some peptides with low molecular weights can cross the intestinal epithelium easily. In general, di- and tri-peptides can be absorbed by H^+^-coupled PepT1.Peptides with a range of four to nine amino acid residues can be successfully transported by the paracellular pathway. Some peptides with 10 amino acid residues and higher are generally believed to be transported by transcytosis.

Several transport enhancers or inhibitors were selected to study the transport mechanisms of QAGLSPVR through the Caco-2 cell monolayer. Gly-Sar is a good substrate for PepT1 and used to evaluate the transport mechanisms of QAGLSPVR. As shown in Figure 3B, Gly-Sar had no significant effect on QAGLSPVR transport (*p* > 0.05), which means QAGLSPVR transport through the Caco-2 cell monolayer is not mediated by PepT1. A previous study indicated that PepT1 is mainly responsible for the transport of di-peptides and tri-peptides but not peptides containing three amino acids and higher [24]. This finding was in accordance with our results. Wortmannin, a transcytos inhibitor, had no significant effect on QAGLSPVR transport (*p* > 0.05), which means QAGLSPVR transport through the Caco-2 cell monolayer is not mediated by transcytosis. In contrast to these substances, cytochalasin D, a disruptor of tight junctions (TJs), significantly increased QAGLSPVR transport (*p* < 0.05), thus revealing that QAGLSPVR could be transported through the Caco-2 cell monolayer via the paracellular pathway. Our results are consistent with those of some food-derived peptides, such as RVPSL [25], QIGLF [8], TNGIIR [18], and GGYR [26].

Quirόs et al. found that LHLPLP is degraded to HLPLP, which shows high antihypertensive effects in animal models [27]. Guo et al. reported that intact RLSFNP and its breakdown fragments F, FNP, SFNP, and RLSF could be detected in the RLSFNP transport solution through the Caco-2 cell monolayer and that RLSFNP fragments, such as FNP, SFNP, and RLSF, contribute to ACEI activities [28]. Therefore, the potential structural changes of QAGLSPVR through the Caco-2 cell monolayer should be evaluated in future studies.

## 4. Materials and Methods

### 4.1. Materials and Reagents

QAGLSPVR was synthesized by Shanghai Synpeptide Co., Ltd. (Shanghai, China). Captopril was purchased from Sinopharm Shantou Jinshi Pharmaceutical Co., Ltd. (Guangdong, China).Caco-2 cells were provided by the Kunming Institute of Zoology (Kunming, China). The ACE activity determination kit used in this work was provided by Shanghai Tongwei Biological Technology Co., Ltd. (Shanghai, China).

### 4.2. Animal Treatment

#### 4.2.1. Animals

Forty-five rats (male, SHRs, SPF; body weight (BW), 240–280 g) were provided by Beijing Vital River Laboratory Animal Technology Co., Ltd. (Beijing, China). The rats were maintained under normal conditions and fed ad libitum under temperature (22 ± 3 °C), humidity (60 ± 5%), and light (12 h light/dark cycle) control. During all animal experiments, strong adherence to International Code of Ethics and National Institutes of Health guidelines for the care and use of laboratory animals was ensured. All rats were divided into three groups (n = 15 per group) after 1 week of feeding; these groups were (1) the control group (normal saline), (2) the positive group (captopril, 10 mg/kg BW dose), and (3) the QAGLSPVR group (QAGLSPVR, 20 mg/kg BW dose).

#### 4.2.2. Measurement of Blood Pressure

The SBP and DBP of the SHRs were measured using the tail-cuff method (IITC Life Science, Woodland, CA., USA) 0, 1, 2, 3, 4, and 5 h after QAGLSPVR administration.

#### 4.2.3. Determination of Serum ACE Activities

Serum was collected from SHRs at 0, 1, 2, 3, 4, and 5 h after intervention, and ACE activities were measured using a kit (Shanghai Tongwei Biological Technology Co., Ltd., Shanghai, China). The determination method strictly complied with the kit instructions.

### 4.3. Transepithelial Transport of QAGLSPVR

Caco-2 cells (1 × 10^5^ cells/well) were routinely cultured in Dublecco’s modified Eagle’s medium with 15% FBS, 100 mg/mL streptomycin and 100 U/mL penicillin. Caco-2 cell monolayer was cultured in six-well Transwell plastic plates in a humidified incubator with 5% CO_2_ at 37 °C and Caco-2 cell monolayer can be used to perform transport experiments when the transepithelial resistance is higher than 400 Ω/cm^2^ [29]. The Caco-2 cell culture medium was substituted with Hank’s balanced salt solution (HBSS) buffer (preheated at 37 °C for 30 min) prior to the transport experiment. The cell monolayer was cultured in HBSS buffer and maintained at 37 °C for 2 h. QAGLSPVR (2 mg/mL, 1.5 mL, dissolved in HBSS) was added to the AP of the cell monolayer, while HBSS buffer (2.5 mL) was added to the BL. The Caco-2 cell monolayer was cultured for 0.5, 1, or 2 h in an incubator at 37 °C. The AP and BL fractions were collected at different times. QAGLSPVR concentrations in the BL were detected by UPLC-Q-Orbitrap-MS^2^ analysis according to our previous method [9], and the transport percentages of QAGLSPVR at different times were calculated.

The transport patterns of QAGLSPVR through the Caco-2 cell monolayer were studied using different transport inhibitors and enhancers [15]. In brief, Gly-Sar, wortmannin, and cytochalasin D were dissolved in DMSO (final concentration of DMSO in HBSS = 0.05%) and final concentrations of25 mM, 500 nM, and 0.5 μg/mL, respectively. The Caco-2 cell monolayer was pre-incubated with Gly-Sar (a peptide transporter PepT1 substrate), wortmannin (atranscytosis inhibitor), or cytochalasin D (a TJ disruptor)for 30 min followed by addition of 1.5 mL of 2 mg/mL QAGLSPVR to the AP and 2.5 mL of fresh HBSS to the BL. After 2 h of incubation, the QAGLSPVR content in the BL was determined via UPLC-Q-Orbitrap-MS^2^ analysis according to our previous method [9], and transport percentages were calculated.

### 4.4. Statistical Analysis

The data were expressed as mean ± standard deviation. Statistical analysis was performed using SPSS software (version 19.0, IBM Inc., Chicago, IL., USA). Differences were considered significant at *p* value < 0.05.

## 5. Conclusions

The antihypertensive effect in vivo of QAGLSPVR derived from tilapia skin gelatin hydrolysates was determined, and the peptide revealed a clear antihypertensive effect on SHRs. The ability of the peptide to inhibit ACE activity in serum was considered a key factor of the antihypertensive effect of QAGLSPVRin vivo. QAGLSPVR could be transported intact by the Caco-2 cell monolayer and may be transported through this layer via the paracellular pathway. Therefore, QAGLSPVR could be effectively absorbed and regulate hypertension in vivo.
